# Association between red blood cell distribution width coefficient of variation and post-treatment bilirubin decline velocity in neonatal hyperbilirubinemia

**DOI:** 10.3389/fped.2025.1690164

**Published:** 2025-12-04

**Authors:** Hongjuan Wei, Tingting Zhang, Rufeng Ji, Yinyan Tang

**Affiliations:** Department of Pediatrics, Nanjing Lishui People's Hospital, Zhongda Hospital Lishui Branch, Southeast University, Nanjing, China

**Keywords:** red blood cell distribution width, neonatal hyperbilirubinemia, bilirubin decline velocity, phototherapy efficacy, hemolytic disease of newborn

## Abstract

**Background:**

Red blood cell distribution width coefficient of variation (RDW-CV) reflects erythrocyte heterogeneity, but its correlation with posttreatment bilirubin kinetics in neonatal hyperbilirubinemia (NHB) remains underexplored.

**Methods:**

This cross-sectional study analyzed 803 neonates (≥35 weeks of gestation) with NHB. RDW-CV was measured at admission, and bilirubin decline velocity was calculated during phototherapy. Associations were evaluated using multivariate linear regression and restricted cubic splines, adjusted for demographic, maternal, and hematologic factors.

**Results:**

Each 1% RDW-CV increase independently reduced bilirubin decline velocity by 2.04 μmol/(L·day) (*β* = −2.04, 95% CI: −3.48∼−0.60, *P* = 0.006). Compared with neonates in the lowest RDW-CV tertile (11.9 to <14.6%), those in the highest tertile (15.2%–20.8%) exhibited a significant reduction in bilirubin decline velocity of 5.33 μmol/(L·day) (*β* = −5.33, 95% CI: −8.44 to −2.21, *P* = 0.001). A linear dose–response trend (*P* = 0.001) was confirmed. Subgroup analyses confirmed consistent associations across neonatal sex, maternal age, and major pregnancy complications (all *P* for interaction > 0.05).

**Conclusions:**

RDW-CV is an independent predictor of bilirubin clearance in NHB, exhibiting a linear dose–response effect. These findings highlight its potential as a biomarker for phototherapy stratification.

## Introduction

Neonatal hyperbilirubinemia (NHB) is a common clinical condition affecting many newborns worldwide (1), with severe cases posing risks of kernicterus and neurodevelopmental impairment (2). Current management practices rely on serial bilirubin measurements to guide phototherapy initiation, yet predictors of treatment efficacy remain inadequately characterized. RDW-CV, a quantitative marker of erythrocyte heterogeneity, has emerged as a potential biomarker for inflammatory and metabolic disorders (3). While elevated RDW-CV correlates with adverse outcomes in cardiovascular and renal diseases (4, 5), its role in neonatal bilirubin clearance remains mechanistically unexplored. Critically, no prior studies have investigated the temporal relationship between RDW-CV and bilirubin clearance kinetics during phototherapy. This study addresses a central question: Is baseline RDW-CV independently associated with posttreatment bilirubin decline velocity in NHB, and how is this relationship modulated by inflammatory, hematologic, and maternal factors? We hypothesize that elevated RDW-CV, reflecting erythropoietic stress and inflammation, impairs bilirubin elimination. Through multivariate linear regression analyses in 803 neonates, we examine the persistence of this association after comprehensive confounder adjustment. These findings establish RDW-CV as a novel predictor of phototherapy responsiveness, potentially informing early risk stratification in NHB management.

## Materials and methods

### Study population

**Figure 1:**
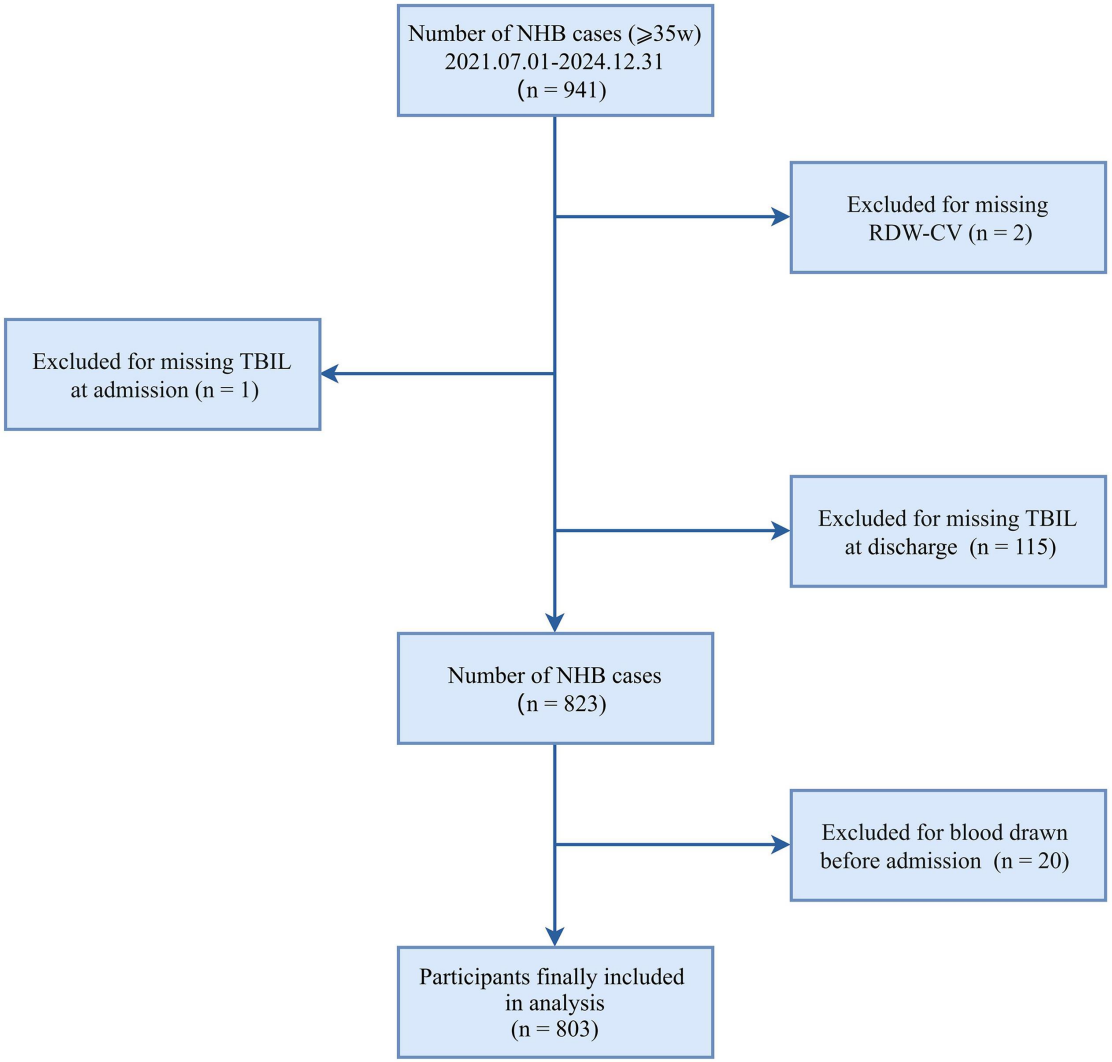
Flowchart for the study population.

This retrospective cross-sectional study enrolled 941 neonates (≥35 weeks of gestation) admitted with hyperbilirubinemia to the neonatal intensive care unit at Nanjing Lishui People's Hospital (NJLSPH) between July 2021 and December 2024. The following exclusion criteria were applied sequentially: missing admission total bilirubin (*n* = 1); absent RDW-CV measurements (*n* = 2); unavailable discharge bilirubin documentation (*n* = 115); and preadmission blood sampling (*n* = 20). The final analytical cohort comprised 803 neonates (85.3% retention; Figure 1). Clinical data were extracted from the Hospital Information System (HIS). The study complied with STROBE guidelines and received institutional ethics approval (Approval Number: 2025KY0703-11), with a waiver of informed consent granted for retrospective analysis of anonymized data.

### Laboratory data collection and measures

Laboratory data were retrospectively gathered from NHB patients admitted to NJLSPH between 1 July 2021 and 31 December 2024. The dataset included demographic information, laboratory test results, and other clinical data. Neonatal demographics comprised sex, birth weight, admission weight, age, blood type, and other relevant factors. Maternal demographics included gestational week, age, blood type, gravidity, parity, and mode of delivery. Laboratory parameters included hematologic indices (WBC, NEUT, LYMPH, RBC, HGB, HCT, RDW-CV, and PLT) and hepatic function markers (TBIL, ALT, AST, GGT, ALP, ALB, and GLO). Clinical biochemical parameters were measured using an automated clinical chemistry analyzer (AU5800, Beckman Coulter Trading Co. Ltd., Shanghai, China). Hematologic indices were assessed using a hematology analyzer (BC-7500, Mindray Corporation, Shenzhen, China).

### Statistical methods

All analyses were performed using R Statistical Software (Version 4.2.2, http://www.R-project.org, The R Foundation) and Free Statistics analysis platform (Version 2.2, Beijing, China, http://www.clinicalscientists.cn/freestatistics). This retrospective cross-sectional study analyzed 803 neonates with hyperbilirubinemia. Baseline characteristics were summarized as mean ± standard deviation, median (interquartile range), or frequency (percentage). Differences across the three RDW-CV tertiles (11.9 to <14.6%, 14.6 to <15.2%, and 15.2% to 20.8%) were assessed using ANOVA or the Kruskal–Wallis test for continuous variables and the chi-square test for categorical variables. Nonlinear relationships were assessed with generalized additive models (GAM) using cubic splines with RDW-CV Winsorized at 0.5th–99.5th percentiles. Missing data in the key covariates of neonatal blood type (*n* = 6 missing), maternal age (*n* = 1 missing), and pre-pregnancy BMI (*n* = 115 missing) were addressed through multiple imputation with chained equations (MICE procedure, five imputations), incorporating all analytic variables to maximize power and minimize selection bias. Regression analyses included (1) univariate linear regression for bilirubin decline velocity [μmol/(L·day)] and (2) multivariate linear regression with hierarchical adjustment, comprising a crude model (unadjusted), Model I (neonatal demographic), Model II (+ maternal factors), and Model III (+ hematologic parameters). As shown in Table 1, for continuous RDW-CV analyses, Model I and Model II utilize a single randomly selected imputed dataset while Model III employs Rubin's rules to pool estimates across all five imputations; categorical RDW-CV analyses uniformly use one imputed dataset. To evaluate the robustness of the association and to assess potential confounding by hemolytic disease of newborn (HDN), a sensitivity analysis (Model IV) was performed by adding the diagnosis of HDN to the fully adjusted Model III. To assess potential unmeasured confounding, the *E*-value was calculated according to the method described by Haneuse et al. (6). Statistical significance was defined as *P* < 0.05. Subgroup analyses were conducted by neonatal sex, WBC, maternal age, GDM, HDP, hypothyroidism, anemia, and prepregnancy BMI using stratified linear regression models, adjusted for covariates consistent with Model III.

**Table 1:** Multivariate linear regression analyses of the associations between RDW-CV and bilirubin decline velocity during treatment for NHB.

RDW-CV category	*N*. total	Non-adjusted Model *β* (95% CI)	*P*	Model Ⅰ *β* (95% CI)	*P*	Model Ⅱ *β* (95% CI)	*P*	Model Ⅲ *β* (95% CI)	*P*
RDW-CV, %	803	−2.68 (−4.02 to −1.35)	<0.001	−2.38 (−3.68 to −1.09)	<0.001	−2.50 (−3.82 to −1.19)	<0.001	−2.04 (−3.48 to −0.60)	0.006
RDW-CV (11.9% −<14.6%)	261	0 (Ref)		0 (Ref)		0 (Ref)		0 (Ref)	
RDW-CV (14.6% −<15.2%)	260	−2.28 (−5.5 to 0.94)	0.165	−1.95 (−5.01 to 1.12)	0.214	−1.66 (−4.73 to 1.41)	0.290	−1.72 (−4.80 to 1.36)	0.275
RDW-CV (15.2%–20.8%)	282	−5.64 (−8.8 to −2.48)	0.001	−5.24 (−8.28 to −2.20)	0.001	−5.21 (−8.29 to −2.14)	0.001	−5.33 (−8.44 to −2.21)	0.001
*P* for trend	803		0.001		0.001		0.001		0.001

Multivariate linear regression analysis of RDW-CV as a continuous variable with bilirubin decline velocity [μmol/(L·day)] in NHB. Non-adjusted model: No covariates were adjusted. Model I: Adjusted for neonatal sex, birth weight, admission weight, neonatal blood type, age at admission, and age at admission blood draw. Model II: Adjusted for model I + maternal age, gestational week, pre-pregnancy BMI, and delivery mode. Model III: Adjusted for model II + WBC, HGB, and PLT.

## Results

### Study design and population

A total of 803 neonates with hyperbilirubinemia were included in the study. The baseline characteristics of the study population are presented in Table 2. Significant differences were observed across the three RDW-CV tertiles in several clinical and laboratory parameters. Specifically, hospitalization duration (*P* < 0.001) and bilirubin decline velocity (*P* = 0.002) were significantly different among the tertiles. Neonates in the highest RDW-CV tertile (15.2%–20.8%) were hospitalized for the longest duration and the exhibited the slowest bilirubin decline velocity [52.2 ± 18.0 μmol/(L·day)]. In addition, significant differences were found in parity (*P* = 0.038), prevalence of GDM (*P* < 0.001), and gestational week (*P* = 0.014) across tertiles. However, no significant differences were observed in sex, birth weight, *Δ*bilirubin, admission TBIL, or discharge TBIL across the tertiles.

**Table 2:** Baseline characteristics of the study population.

Variable	Total (*n* = 803)	RDW-CV tertile 1 (11.9% to <14.6%) (*n* = 261)	RDW-CV tertile 2 (14.6% to <15.2%) (*n* = 260)	RDW-CV tertile 3 (15.2% to 20.8%) (*n* = 282)	*P*
Diagnoses					<0.001
NHB	701 (87.3)	240 (92.0)	232 (89.2)	229 (81.2)	
NHB (HDN)	102 (12.7)	21 (8.0)	28 (10.8)	53 (18.8)	
Sex, *n* (%)					0.160
Male	431 (53.7)	128 (49.0)	142 (54.6)	161 (57.1)	
Female	372 (46.3)	133 (51.0)	118 (45.4)	121 (42.9)	
Birth weight, g	3,357.6 ± 425.3	3,358.6 ± 385.1	3,364.6 ± 424.6	3,350.4 ± 461.3	0.927
Admission weight, g	3,207.5 ± 425.4	3,239.3 ± 385.4	3,215.2 ± 436.0	3,171.0 ± 448.9	0.164
Neonatal blood type, *n* (%)					0.356
O+	224 (28.1)	79 (30.6)	74 (28.5)	71 (25.4)	
A+	276 (34.6)	86 (33.3)	81 (31.2)	109 (39.1)	
B+	237 (29.7)	73 (28.3)	81 (31.2)	83 (29.7)	
AB+	58 (7.3)	20 (7.8)	23 (8.8)	15 (5.4)	
O−	1 (0.1)	0 (0)	1 (0.4)	0 (0)	
AB−	1 (0.1)	0 (0)	0 (0)	1 (0.4)	
hsCRP, mg/L	1.0 (0.5, 3.1)	0.8 (0.3, 2.2)	1.2 (0.5, 3.6)	1.5 (0.8, 3.6)	<0.001
RBC, 10^12^/L	4.9 ± 0.6	4.9 ± 0.5	4.9 ± 0.6	5.0 ± 0.7	0.016
HGB, g/L	172.6 ± 20.2	170.1 ± 18.3	172.7 ± 20.2	174.9 ± 21.6	0.020
HCT, %	49.6 ± 6.1	49.1 ± 5.7	49.7 ± 6.2	49.8 ± 6.3	0.364
RDW-CV, %	15.0 ± 1.0	14.1 ± 0.3	14.8 ± 0.2	16.0 ± 0.9	<0.001
WBC, 10^9^/L	10.5 ± 2.9	10.9 ± 2.4	10.6 ± 2.8	10.2 ± 3.3	0.026
NEUT, 10^9^/L	4.8 ± 2.2	4.8 ± 1.9	4.8 ± 2.2	4.7 ± 2.4	0.676
LYMPH, 10^9^/L	4.1 ± 1.4	4.4 ± 1.3	4.1 ± 1.5	4.0 ± 1.4	0.004
MONO, 10^9^/L	1.1 ± 0.4	1.2 ± 0.4	1.1 ± 0.4	1.0 ± 0.4	<0.001
PLT, 10^9^/L	286.4 ± 81.2	300.7 ± 76.9	288.1 ± 80.6	271.6 ± 83.4	<0.001
PCT, %	0.3 ± 0.1	0.3 ± 0.1	0.3 ± 0.1	0.3 ± 0.1	0.365
ALT, U/L	16.5 ± 6.2	16.6 ± 5.5	16.8 ± 7.1	16.3 ± 5.9	0.601
AST, U/L	45.9 ± 24.7	42.2 ± 14.0	47.8 ± 34.8	47.6 ± 20.6	0.014
ALB, g/L	36.1 ± 3.2	36.2 ± 3.1	36.2 ± 3.1	36.0 ± 3.3	0.529
GLO, g/L	23.4 ± 3.8	23.6 ± 3.9	23.7 ± 3.8	23.1 ± 3.7	0.108
A/G	1.6 ± 0.3	1.6 ± 0.4	1.6 ± 0.3	1.6 ± 0.3	0.741
GGT, U/L	152.8 ± 68.5	145.0 ± 62.5	153.4 ± 71.4	159.3 ± 70.7	0.052
ALP, U/L	162.5 ± 54.1	160.9 ± 53.4	162.1 ± 50.1	164.5 ± 58.2	0.720
GLU, mmol/L	4.2 (3.6, 4.8)	4.3 (3.8, 4.9)	4.2 (3.5, 4.8)	4.2 (3.5, 4.8)	0.108
CK-MB, U/L	39.3 ± 18.2	38.9 ± 16.1	39.6 ± 16.5	39.5 ± 21.3	0.869
K^+^, mmol/L	4.9 ± 0.5	4.9 ± 0.5	5.0 ± 0.5	4.8 ± 0.5	0.007
Na^+^, mmol/L	139.5 ± 3.0	139.0 ± 2.5	139.5 ± 3.0	140.0 ± 3.3	< 0.001
Cl^−^, mmol/L	106.8 ± 3.2	106.7 ± 3.0	106.7 ± 3.2	107.1 ± 3.4	0.251
Admission TBIL, μmol/L	305.9 ± 49.6	304.5 ± 45.8	302.3 ± 47.7	310.4 ± 54.3	0.139
Discharge TBIL, μmol/L	140.8 ± 27.7	139.2 ± 25.0	140.9 ± 26.8	142.2 ± 30.7	0.443
*Δ*Bilirubin, μmol/L	165.1 ± 50.2	165.3 ± 47.8	161.3 ± 50.5	168.2 ± 52.0	0.277
Age at admission, h	130.3 ± 77.8	141.1 ± 79.3	128.9 ± 77.0	121.7 ± 76.2	0.014
Age at admission blood draw, h	131.2 ± 77.8	141.9 ± 79.3	129.9 ± 77.1	122.6 ± 76.2	0.015
Hospitalization duration, h	96.4 ± 20.4	91.8 ± 19.1	94.1 ± 18.4	102.8 ± 21.7	<0.001
The time interval between blood sampling at admission and discharge, h	75.3 ± 18.7	71.2 ± 17.0	73.0 ± 16.9	81.3 ± 20.2	<0.001
Bilirubin decline velocity, μmol/(L·h)	2.3 ± 0.8	2.4 ± 0.7	2.3 ± 0.9	2.2 ± 0.7	0.002
Bilirubin decline velocity, μmol/(L·day)	55.1 ± 18.9	57.8 ± 17.7	55.5 ± 20.6	52.2 ± 18.0	0.002
Maternal age, year	29.9 ± 4.6	29.7 ± 4.3	30.4 ± 4.6	29.7 ± 4.8	0.168
Education, *n* (%)					0.566
Elementary school	12 (1.7)	4 (1.9)	4 (1.8)	4 (1.5)	
Junior high school	92 (13.4)	24 (11.6)	28 (12.8)	40 (15.3)	
High school	50 (7.3)	16 (7.7)	13 (6.0)	21 (8.0)	
Junior college	68 (9.9)	17 (8.2)	19 (8.7)	32 (12.3)	
College	217 (31.6)	63 (30.4)	71 (32.6)	83 (31.8)	
Bachelor	224 (32.7)	76 (36.7)	78 (35.8)	70 (26.8)	
Master’s degree or above	23 (3.4)	7 (3.4)	5 (2.3)	11 (4.2)	
Gestational week, week	39.0 ± 1.2	39.1 ± 1.2	39.1 ± 1.2	38.8 ± 1.3	0.014
Gravidity, *n* (%)					0.171
1	375 (46.7)	135 (51.7)	126 (48.5)	114 (40.4)	
2	187 (23.3)	58 (22.2)	59 (22.7)	70 (24.8)	
3	123 (15.3)	38 (14.6)	37 (14.2)	48 (17.0)	
4	63 (7.8)	18 (6.9)	18 (6.9)	27 (9.6)	
5	36 (4.5)	8 (3.1)	16 (6.2)	12 (4.3)	
≥6	19 (2.4)	4 (1.5)	4 (1.5)	11 (3.9)	
Parity, *n* (%)					0.038
1	475 (59.2)	167 (64.0)	157 (60.4)	151 (53.5)	
2	278 (34.6)	78 (29.9)	91 (35.0)	109 (38.7)	
3	45 (5.6)	15 (5.7)	9 (3.5)	21 (7.4)	
4	4 (0.5)	0 (0)	3 (1.2)	1 (0.4)	
5	1 (0.1)	1 (0.4)	0 (0)	0 (0)	
Delivery mode, *n* (%)					0.004
Vaginal delivery	506 (63.0)	186 (71.3)	161 (61.9)	159 (56.4)	
Cesarean section	282 (35.1)	71 (27.2)	93 (35.8)	118 (41.8)	
Vaginal delivery to cesarean section	15 (1.9)	4 (1.5)	6 (2.3)	5 (1.8)	
Maternal blood type, *n* (%)					0.293
O+	295 (39.5)	77 (34.2)	97 (39.9)	121 (43.4)	
A+	200 (26.8)	65 (28.9)	58 (23.9)	77 (27.6)	
B+	196 (26.2)	67 (29.8)	66 (27.2)	63 (22.6)	
AB+	53 (7.1)	15 (6.7)	20 (8.2)	18 (6.5)	
O−	3 (0.4)	1 (0.4)	2 (0.8)	0 (0)	
Pre-pregnancy BMI, kg/m^2^	22.6 ± 3.6	21.9 ± 3.4	22.5 ± 3.5	23.1 ± 3.7	0.002
GDM, *n* (%)					<0.001
No	619 (77.1)	220 (84.3)	213 (81.9)	186 (66.0)	
Yes	184 (22.9)	41 (15.7)	47 (18.1)	96 (34.0)	
HDP, *n* (%)					0.115
No	739 (92.0)	245 (93.9)	242 (93.1)	252 (89.4)	
Yes	64 (8.0)	16 (6.1)	18 (6.9)	30 (10.6)	
Hypothyroidism, *n* (%)					0.165
No	735 (91.5)	243 (93.1)	241 (92.7)	251 (89.0)	
Yes	68 (8.5)	18 (6.9)	19 (7.3)	31 (11.0)	
ICP, *n* (%)					0.605
No	787 (98.0)	257 (98.5)	253 (97.3)	277 (98.2)	
Yes	16 (2.0)	4 (1.5)	7 (2.7)	5 (1.8)	
Anemia, *n* (%)					0.764
No	744 (92.7)	244 (93.5)	241 (92.7)	259 (91.8)	
Yes	59 (7.3)	17 (6.5)	19 (7.3)	23 (8.2)	

Data are presented as mean ± standard deviation (SD) for normally distributed continuous variables, median (interquartile range, IQR) for skewed continuous variables, and number (percentage) for categorical variables. *Δ*Bilirubin denotes the reduction in total bilirubin concentration between admission and discharge. Continuous variables were compared using one-way ANOVA (normal distribution) or Kruskal–Wallis test (non-normal distribution); categorical variables were compared with *χ*^2^ test or Fisher's exact test as appropriate. Missing data were present for the following variables: neonatal blood type (*n* = 6), maternal education (*n* = 117), maternal blood type (*n* = 56), pre-pregnancy BMI (*n* = 115), hsCRP (*n* = 16), ALT (*n* = 2), AST (*n* = 2), ALB (*n* = 2), GLO (*n* = 2), A/G (*n* = 2), GGT (*n* = 2), ALP (*n* = 2), CK-MB (*n* = 2), GLU (*n* = 208), K^+^ (*n* = 3), Na^+^ (*n* = 3), and Cl^−^ (*n* = 3). Analyses for these variables are based on the available cases.

### Nonlinear relationships

**Figure 2:**
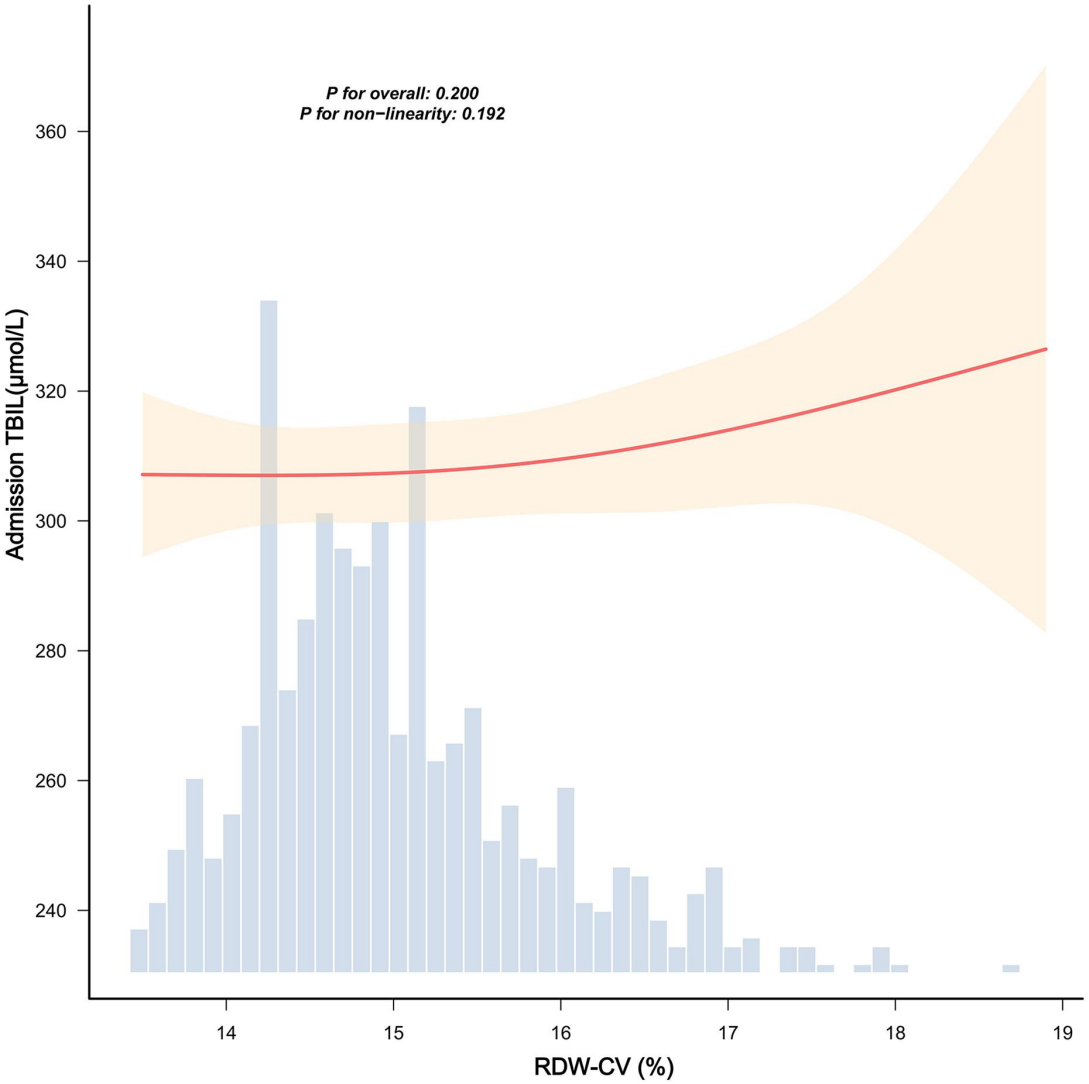
Smooth curve fittings between RDW-CV and admission TBIL. The red solid line indicates the linear regression fit between RDW-CV (%) and admission TBIL (μmol/L), representing the estimated linear relationship. The shaded yellow band indicates the 95% confidence interval around the regression line. The blue histogram represents the frequency distribution of RDW-CV values across the study population. The RDW-CV range was truncated at the 0.5th–99.5th percentiles to exclude extreme values. The linear regression was adjusted for neonatal sex, birth weight, admission weight, age at admission, age at admission blood draw, maternal age, gestational week, prepregnancy BMI, delivery mode, WBC, HGB, and PLT. *P*-values denote the overall association (*P* = 0.200) and nonlinearity test (*P* = 0.192).

**Figure 3:**
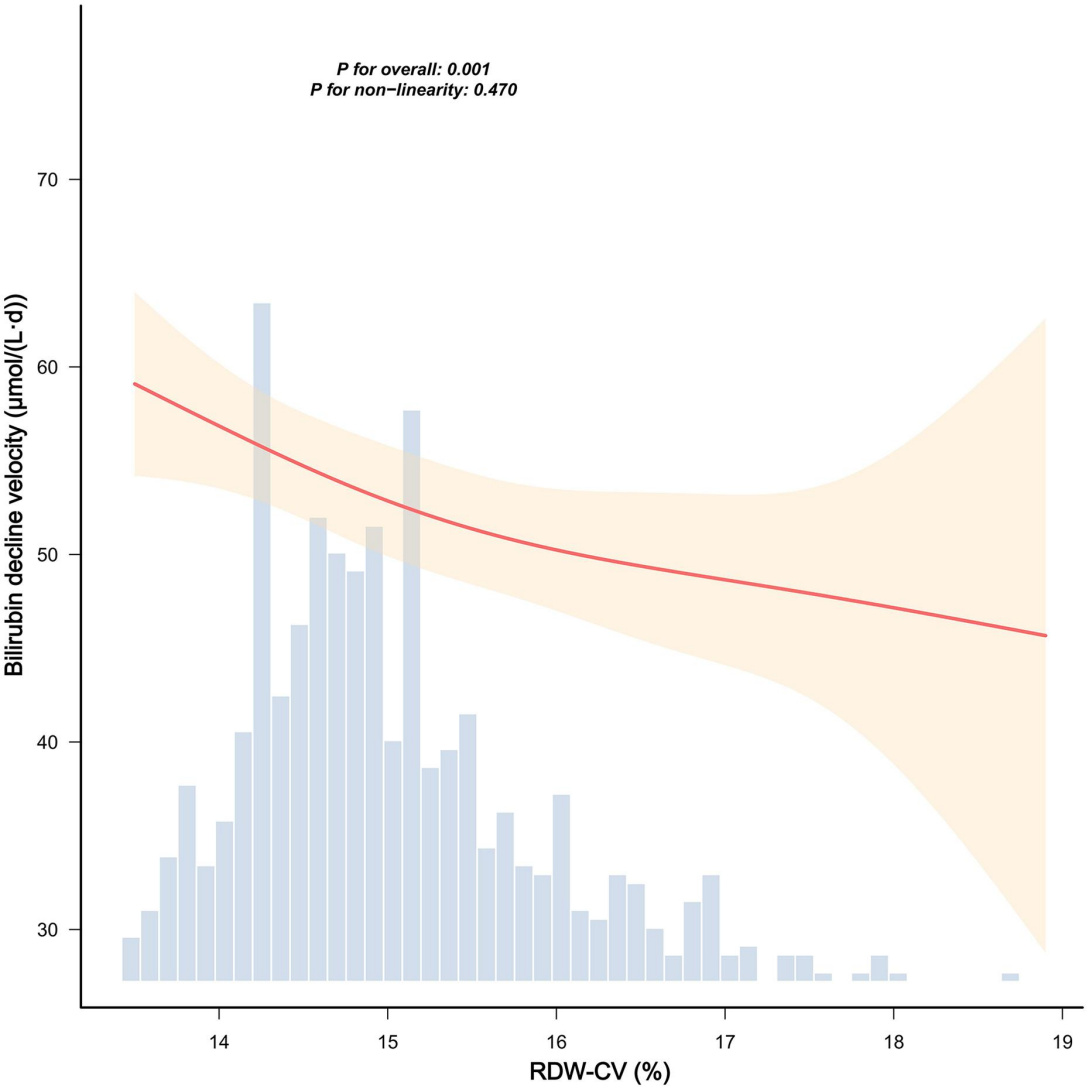
Smooth curve fittings between RDW-CV and bilirubin decline velocity. The red solid line indicates the smooth curve fit between RDW-CV (%) and bilirubin decline velocity [μmol/(L·day)], representing the adjusted association. The shaded yellow band indicates the 95% confidence interval around the curve. The blue histogram represents the frequency distribution of RDW-CV values across the study population. The RDW-CV range was truncated at the 0.5th–99.5th percentiles to exclude extreme values. The smooth curve fitting was adjusted for neonatal sex, birth weight, admission weight, age at admission, age at admission blood draw, maternal age, gestational week, prepregnancy BMI, delivery mode, WBC, HGB, and PLT. *P*-values denote the overall association (*P* = 0.001) and nonlinearity test (*P* = 0.470), indicating that the relationship between RDW-CV and bilirubin decline velocity is statistically significant overall, but the nonlinearity is not significant.

Figure 2 demonstrates no significant association between RDW-CV and admission bilirubin levels (*P* for overall = 0.200; *P* for nonlinearity = 0.192). Conversely, Figure 3 reveals a statistically significant inverse linear relationship between higher RDW-CV and slower bilirubin decline velocity (*P* for overall = 0.001; *P* for nonlinearity = 0.470), indicating a linear association between these variables.

### Regression analyses section

**Figure 4:**
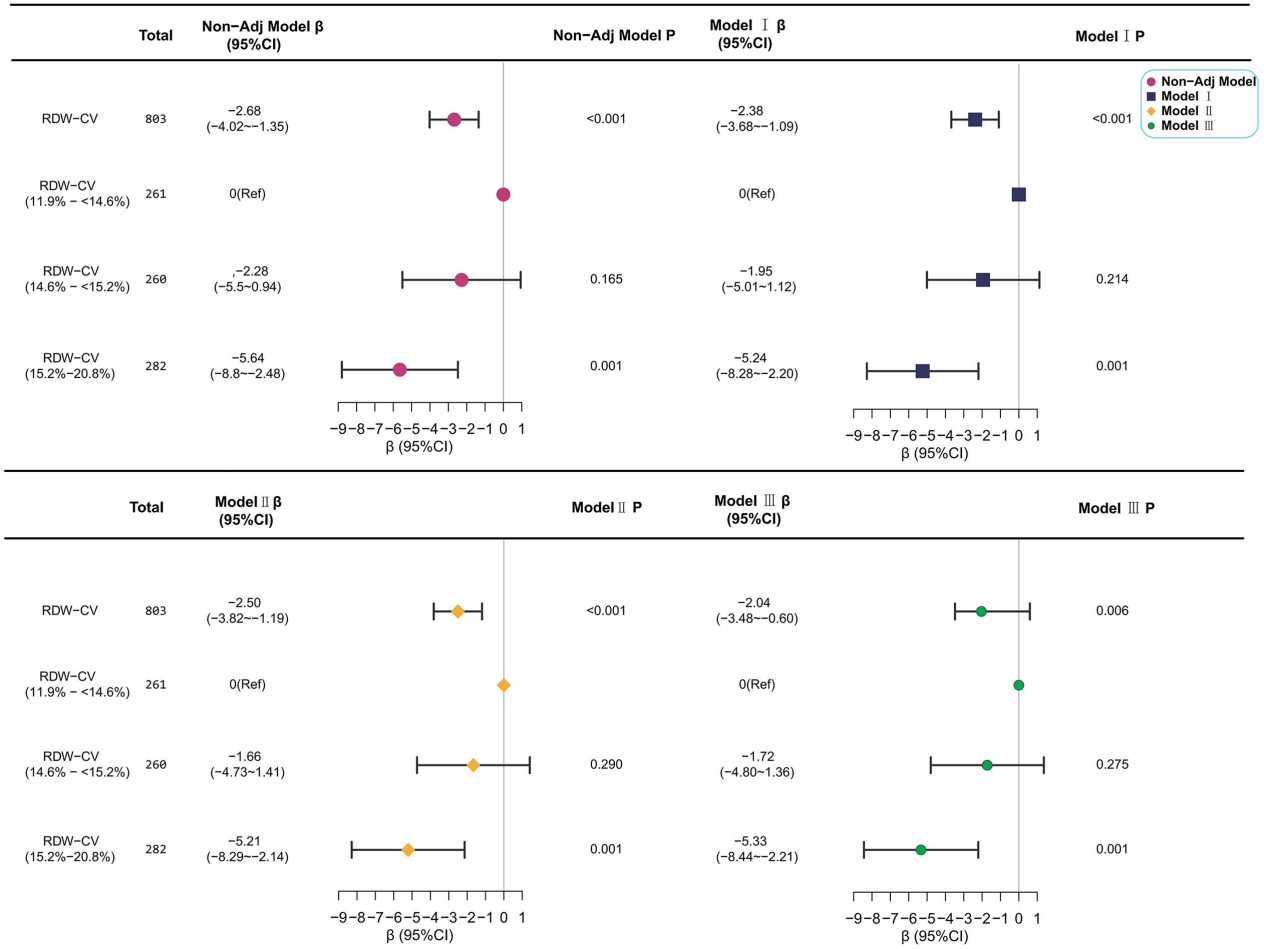
Forest plot of analyses for the association between RDW-CV and bilirubin decline velocity during treatment for NHB. Symbols represent regression coefficients (*β*) with 95% confidence intervals (horizontal lines): Solid pink-purple circle for the non-adjusted model, blue square for Model I, yellow diamond for Model II, and solid green circle for Model III. Model adjustment covariates are consistent with Table 1. The vertical dashed line (*β* = 0) indicates no effect. For categorical RDW-CV (%), the reference group is 11.9% to  < 14.6%. Bilirubin decline velocity is expressed in μmol/(L·day).

Univariate regression identified RDW-CV as a significant independent predictor of reduced bilirubin clearance velocity [*β* = −2.68 μmol/(L·day), 95% *CI*: −4.02 to −1.35, *P* < 0.001; Table 3]. Other significant indicators in univariate analysis included elevated hs-CRP (*β* = −0.52), NEUT (*β* = −1.44), and LYMPH (*β* = 3.70) (all *P* < 0.001). Furthermore, a higher  admission TBIL predicted a faster decline (*β* = 0.20, *P* < 0.001), whereas a longer hospitalization duration was associated with a slower decline (*β* = −0.41, *P* < 0.001). In multivariate analyses, this inverse association between RDW-CV and bilirubin decline velocity persisted robustly after sequential adjustment for demographic, maternal, and hematologic confounders. For continuous RDW-CV, each 1% increase corresponded to a 2.04 μmol/(L·day) reduction in clearance velocity in the fully adjusted model (*β* = −2.04, 95% CI: −3.48 to −0.60; *P* = 0.006). Categorically, neonates in the highest RDW-CV tertile (15.2%–20.8%) demonstrated a significantly slower decline than those in the lowest tertile [*β* = −5.33 μmol/(L·day), 95% CI: −8.44 to −2.21; *P* = 0.001], with a pronounced dose–response relationship (*P* for trend = 0.001) (Table 1, Figure 4). Critically, the association remained significant in a sensitivity analysis (Model IV) that adjusted for HDN, with consistent results for both continuous RDW-CV (*β* = −2.27, 95% CI: −3.60 to −0.94; *P* = 0.001) and the highest tertile (*β* = −4.68, 95% CI: −7.79 to −1.57; *P* = 0.003), confirming its independence from overt hemolysis (Supplementary Table S1).

**Table 3:** Univariate linear regression analysis of factors associated with bilirubin decline velocity during treatment for NHB.

Variable	*β* (95% CI)	*P* (*t*-test)	*P* (*F*-test)
Sex, *n* (%)			0.555
Male	(Ref)		
Female	0.79 (−1.83, 3.41)	0.555	
Birth weight, per 100 g	0.17 (−0.14, 0.48)	0.277	0.277
Admission weight, per 100 g	0.08 (−0.22, 0.39)	0.594	0.594
Neonatal blood type, *n* (%)			0.064
O+	(Ref)		
A+	−0.38 (−3.70, 2.95)	0.825	
B+	−0.79 (−4.23, 2.65)	0.653	
AB+	4.36 (−1.08, 9.80)	0.116	
O−	−47.86 (−84.85, −10.88)	0.011	
AB−	−11.23 (−48.21, 25.76)	0.551	
Hs-CRP, mg/L	−0.52 (−0.81, −0.22)	< 0.001	<0.001
RBC, 10^12^/L	0.84 (−1.34, 3.02)	0.450	0.450
HGB, g/L	−0.01 (−0.08, 0.05)	0.694	0.694
HCT, %	−0.05 (−0.26, 0.17)	0.677	0.677
RDW-CV, %	−2.68 (−4.02, −1.35)	< 0.001	<0.001
WBC, 10^9^/L	0.13 (−0.33, 0.58)	0.580	0.580
NEUT, 10^9^/L	−1.44 (−2.03, −0.85)	< 0.001	<0.001
LYMPH, 10^9^/L	3.70 (2.80, 4.61)	< 0.001	<0.001
MONO, 10^9^/L	5.16 (1.96, 8.36)	0.002	0.002
PLT, 10^9^/L	0.03 (0.01, 0.05)	< 0.001	<0.001
PCT, %	33.91 (18.17, 49.64)	< 0.001	<0.001
ALT, U/L	0.17 (−0.04, 0.38)	0.110	0.110
AST, U/L	−0.07 (−0.12, −0.01)	0.014	0.014
ALB, g/L	1.34 (0.93, 1.74)	< 0.001	<0.001
GLO, g/L	0.30 (−0.04, 0.65)	0.088	0.088
A/G	3.37 (−0.40, 7.13)	0.079	0.079
GGT, U/L	−0.01 (−0.03, 0.01)	0.315	0.315
ALP, U/L	0.03 (0.01, 0.06)	0.005	0.005
GLU, mmol/L	−0.08 (−0.42, 0.27)	0.659	0.659
CK-MB, U/L	0.05 (−0.02, 0.12)	0.171	0.171
K^+^, mmol/L	6.39 (3.93, 8.86)	< 0.001	<0.001
Na^+^, mmol/L	−0.13 (−0.57, 0.31)	0.565	0.565
Cl^−^, mmol/L	−0.77 (−1.17, −0.36)	< 0.001	<0.001
Admission TBIL, μmol/L	0.20 (0.18, 0.23)	< 0.001	<0.001
Discharge TBIL, μmol/L	−0.13 (−0.18, −0.08)	< 0.001	<0.001
Age at admission, h	0.06 (0.05, 0.08)	< 0.001	<0.001
Age at admission blood draw, h	0.06 (0.05, 0.08)	< 0.001	<0.001
Hospitalization duration, h	−0.41 (−0.47, −0.36)	< 0.001	<0.001
Maternal age, year	−0.34 (−0.62, −0.05)	0.020	0.020
Education, *n* (%)			0.071
Elementary school	(Ref)		
Junior high school	1.30 (−10.19, 12.79)	0.824	
High school	10.08 (−1.96, 22.11)	0.101	
Junior college	8.07 (−3.65, 19.79)	0.177	
College	2.68 (−8.42, 13.79)	0.635	
Bachelor	3.78 (−7.32, 14.87)	0.504	
Master’s degree or above	5.03 (−8.30, 18.37)	0.459	
Gestational week, week	−0.01 (−1.06, 1.04)	0.986	0.986
Gravidity, *n* (%)			0.115
1	(Ref)		
2	−1.73 (−5.04, 1.57)	0.304	
3	−2.76 (−6.60, 1.08)	0.159	
4	4.54 (−0.49, 9.57)	0.077	
5	−4.42 (−10.87, 2.02)	0.178	
≥6	−1.66 (−10.35, 7.03)	0.708	
Parity, *n* (%)			0.965
1	(Ref)		
2	0.14 (−2.66, 2.94)	0.923	
3	0.30 (−5.49, 6.08)	0.920	
4	5.31 (−13.32, 23.94)	0.576	
5	9.70 (−27.45, 46.85)	0.608	
Delivery mode, *n* (%)			0.796
Vaginal delivery	(Ref)		
Cesarean section	0.60 (−2.16, 3.35)	0.671	
Vaginal delivery to cesarean section	−2.37 (−12.08, 7.34)	0.632	
Maternal blood type, *n* (%)			<0.001
O+	(Ref)		
A+	5.15 (1.79, 8.52)	0.003	
B+	8.08 (4.70, 11.47)	<0.001	
AB+	4.96 (−0.52, 10.44)	0.076	
O−	−13.96 (−35.28, 7.36)	0.199	
Prepregnancy BMI, kg/m^2^	−0.03 (−0.43, 0.37)	0.871	0.871

Bilirubin decline velocity is expressed in μmol/(L·day). Data are presented as *β* and 95% CI*.* For birth weight and admission weight, *β* coefficients represent the change in bilirubin decline velocity per 100 g increase in weight. *P*-values are identical to those from the original per-gram analysis as linear transformations do not alter statistical significance. All analyses in Table 2 are based on the original dataset without imputation.

### Subgroup analyses

**Figure 5:**
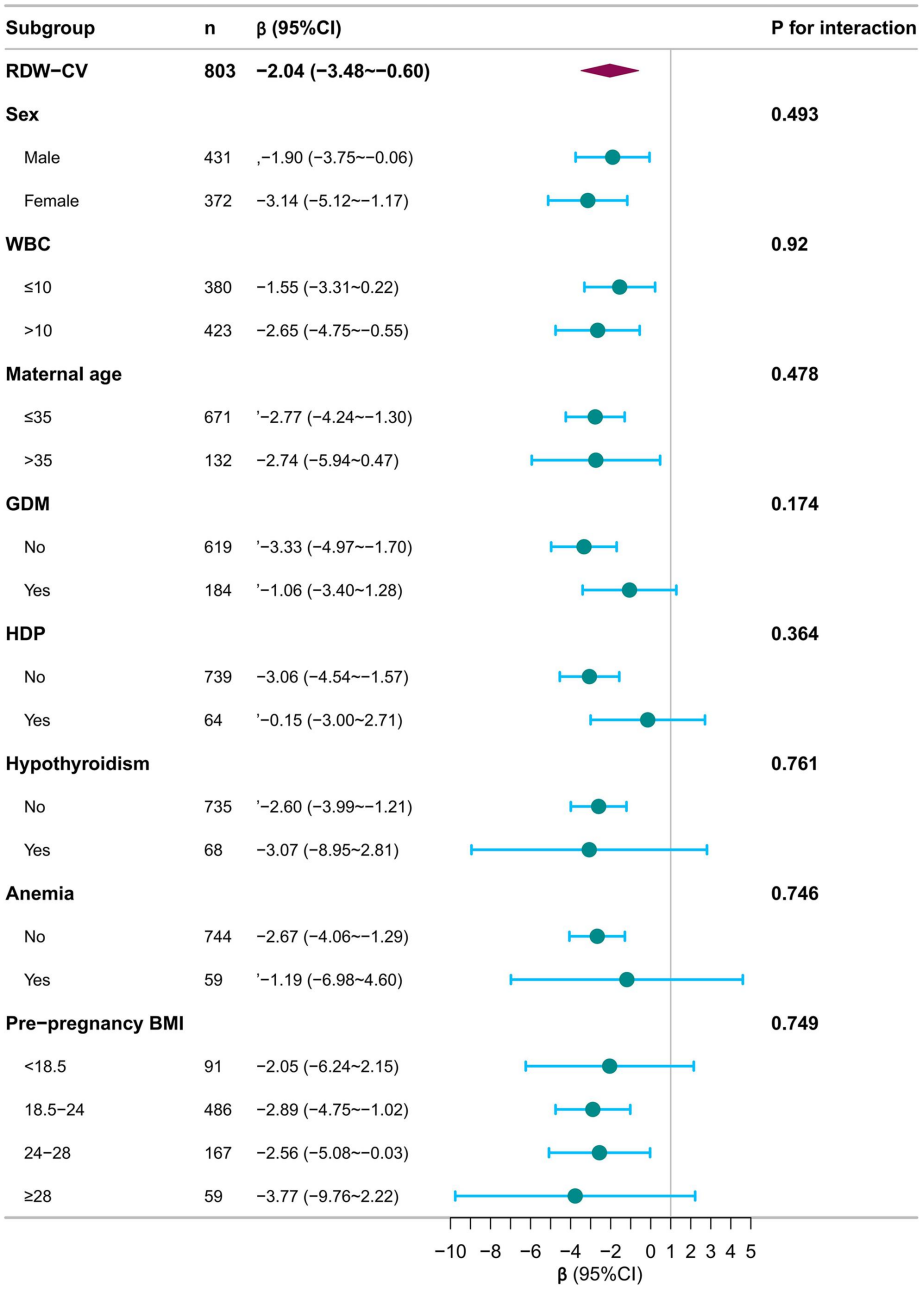
Forest plot of subgroup analyses for the association between RDW-CV and bilirubin decline velocity during treatment for NHB. Forest plot shows regression coefficients (95% CI) for the association between RDW-CV and bilirubin decline velocity. Model adjusted for covariates consistent with Model III. Bilirubin decline velocity is expressed in μmol/(L·day).

Subgroup analyses revealed consistent effects across all clinically relevant strata. The inverse association was robust in both males (*β* = −1.90) and females (*β* = −3.14), in neonates with (*β* = −1.06) and without (*β* = −3.33) GDM, and across different strata of maternal age, WBC count, and other pregnancy complications (Table 4). No significant interactions were observed (all *P* for interaction >0.05), visually confirmed by the forest plot in Figure 5.

**Table 4:** Subgroup analysis of the relationship between RDW-CV and bilirubin decline velocity during treatment for NHB.

Subgroup	*N*	*β* (95% CI)	*P* for interaction
RDW-CV, %	803	−2.04 (−3.48 to −0.60)	
Sex			0.493
Male	431	−1.90 (−3.75 to −0.06)	
Female	372	−3.14 (−5.12 to −1.17)	
WBC, 10^9^/L			0.920
≤10	380	−1.55 (−3.31 to 0.22)	
>10	423	−2.65 (−4.75 to −0.55)	
Maternal age, years			0.478
≤35	671	−2.77 (−4.24 to −1.30)	
>35	132	−2.74 (−5.94 to 0.47)	
GDM			0.174
No	619	−3.33 (−4.97 to −1.70)	
Yes	184	−1.06 (−3.40 to 1.28)	
HDP			0.364
No	739	−3.06 (−4.54 to −1.57)	
Yes	64	−0.15 (−3.00 to 2.71)	
Hypothyroidism			0.761
No	735	−2.60 (−3.99 to −1.21)	
Yes	68	−3.07 (−8.95 to 2.81)	
Anemia			0.746
No	744	−2.67 (−4.06 to −1.29)	
Yes	59	−1.19 (−6.98 to 4.60)	
Prepregnancy BMI, kg/m^2^			0.749
<18.5	91	−2.05 (−6.24 to 2.15)	
18.5–24	486	−2.89 (−4.75 to −1.02)	
24–28	167	−2.56 (−5.08 to −0.03)	
≥28	59	−3.77 (−9.76 to 2.22)	

Model adjusted for covariates consistent with Model III. Bilirubin decline velocity is expressed in μmol/(L·day).

## Discussion

### Principal findings

This study establishes RDW-CV as an independent predictor of bilirubin clearance kinetics in NHB. We demonstrate that (1) each 1% RDW-CV increase reduces bilirubin decline velocity by 2.04 μmol/(L·day) after comprehensive adjustment. (2) A linear dose–response relationship exists (*P* for trend = 0.001), with neonates in the highest RDW-CV tertile (15.2%–20.8%) exhibiting 5.33 μmol/(L·day) slower clearance than those in the lowest tertile (11.9 to  < 14.6%). (3) Subgroup analyses confirmed robust consistency across pregnancy complications, hematologic profiles, and maternal age groups (all *P* for interaction > 0.05). (4) This association is specific to treatment response rather than baseline severity, as evidenced by null admission bilirubin correlation (*P* = 0.200).

### Other findings

Given the established links between RBC and NHB (7), we specifically investigated the relationship between RDW-CV and other RBC counts. A statistically significant, positive association was observed, which remained after multivariate adjustment (Supplementary Table S2). However, this association was weak, as indicated by a minimal effect size (*β* = 0.03 per 1% RDW-CV increase), and was sensitive to extreme values, becoming non-significant upon their exclusion (Supplementary Figure S1). Therefore, the biological and clinical significance of this relationship remains uncertain and warrants further investigation in future studies.

### Comparison with existing literature

We observed a significantly higher prevalence of HDN in the highest RDW-CV tertile group (Table 2). To determine whether the association between RDW-CV and bilirubin decline velocity was independent of the hemolytic process, we performed a critical additional adjustment for the diagnosis of HDN in the multivariate model (Model IV). After this adjustment, the significant negative association persisted (Supplementary Table S1), indicating that the predictive value of RDW-CV extends beyond hemolysis and may reflect broader pathophysiological mechanisms, such as systemic inflammation or oxidative stress, which can independently influence bilirubin metabolism and clearance.

Our findings reveal a novel inverse linear relationship between RDW-CV and bilirubin clearance velocity in NHB, in contrast with prior literature in three key aspects. First, while elevated RDW-CV is well established in hemolytic hyperbilirubinemia (ABO/Rh incompatibility, G6PD deficiency) as a marker of bilirubin production (8, 9), no previous studies link RDW-CV to bilirubin elimination kinetics. Most neonatal research focuses on the diagnostic utility of RDW-CV for hemolysis (10), not posttreatment dynamics. Second, studies with adults associate high RDW-CV with impaired liver function in cholestatic diseases (11–13). However, neonatal bilirubin metabolism differs fundamentally in UGT1A1 enzyme maturity and transporter expression (14), limiting direct extrapolation. Third, the observed absence of significant association between RDW-CV and bilirubin measures aligns with neonatal studies (15), yet diverges from investigations specifically focused on hemolytic etiologies where RDW-CV elevation reflects active erythrocyte destruction (16). This underscores the distinct role of RDW-CV in bilirubin clearance vs. bilirubin load. Notably, our robust dose–response relationship [*β* = −2.04 μmol/(L·day), *P* = 0.006] persists despite adjusting for key confounders absent in prior neonatal RDW analyses.

### Mechanistic interpretation of linear association

The correlation between increased RDW-CV and impaired bilirubin clearance in NHB may be mediated through several pathophysiological pathways. First, increased RDW-CV reflects greater heterogeneity in RBC size (17), which is strongly associated with ineffective erythropoiesis and accelerated hemolysis (18). This leads to excessive release of hemoglobin-derived heme, overwhelming the neonatal liver's capacity for bilirubin conjugation and excretion, thereby contributing to sustained hyperbilirubinemia (19). Second, elevated RDW-CV is closely linked to systemic inflammation and oxidative stress (20, 21), both of which are known to suppress the activity of UDP-glucuronosyltransferase 1A1 (UGT1A1) (22, 23), the rate-limiting enzyme responsible for bilirubin conjugation. Proinflammatory cytokines such as IL-6 may further impair hepatic bilirubin metabolism by disrupting cellular signaling pathways essential for UGT1A1 expression and function (24–26). Third, patients with high RDW-CV often exhibit hepatic immaturity or dysfunction, characterized by reduced enzymatic activity, which diminishes the liver's ability to efficiently process and excrete bilirubin (27). Finally, the presence of abnormal RBC morphology associated with elevated RDW-CV can lead to microcirculatory disturbances, impairing hepatic perfusion and further delaying bilirubin clearance (28, 29). The interplay of these mechanisms suggests that elevated RDW-CV may serve as a valuable biomarker for predicting treatment response in NHB, potentially guiding clinical management decisions.

### Strengths and limitations

This study offers novel insights into the relationship between RDW-CV and bilirubin kinetics in NHB by quantifying its dose–response effect. The use of spline regression further confirms the linear relationship between RDW-CV and bilirubin decline velocity. In addition, the robustness of these findings is supported by comprehensive subgroup analyses across various clinical strata, including neonatal sex, maternal age, and major pregnancy complications. However, the study has several limitations. First, due to its cross-sectional design, we cannot establish temporal relationships or definitively prove causality between RDW-CV and bilirubin decline velocity. Second, the single-center design limits generalizability, although our sample (*n* = 803) exceeds that of similar studies (8). Third, residual confounding from unmeasured factors cannot be excluded despite adjustments. However, the large *E*-value (12.28) for the association (Model III *β* = −2.04) suggests that it is unlikely to be fully explained by confounding. These limitations should be considered when interpreting the results.

## Conclusions

This study demonstrates that RDW-CV is independently associated with bilirubin clearance velocity in NHB, exhibiting a clear linear dose–response relationship. These findings highlight the potential value of RDW-CV as a biomarker for assessing phototherapy efficacy in clinical practice. Future research should aim to replicate these results in larger, multicenter studies and elucidate the mechanisms underlying the association between RDW-CV and bilirubin kinetics.

## Data Availability

The original contributions presented in the study are included in the article/Supplementary Material; further inquiries can be directed to the corresponding author.
