# Supplementary material for: Association between red blood cell distribution width coefficient of variation and post-treatment bilirubin decline velocity in neonatal hyperbilirubinemia
Source: Front Pediatr. 2025 Dec 4;13:1690164. doi: 10.3389/fped.2025.1690164 (PMC12711753; doi:10.3389/fped.2025.1690164)
Supplement: Supplementary file 1 [file Table1.doc]

**Title: Association Between Red Blood Cell Distribution Width Coefficient of Variation and Post Treatment Bilirubin Decline Velocity in Neonatal Hyperbilirubinemia**

**Supplementary Materials**

**Supplementary Figure 1. Smooth curve fittings between RDW-CV(%) and admission RBC count (*10^12^/L).**

**Supplementary Table 1. Multivariable linear regression analyses of the associations between RDW-CV and **bilirubin decline velocity during treatment in NHB.****

**Supplementary Table 2. Multivariable linear regression analyses of the associations between RDW-CV and **RBC** count**.****

****Supplementary Figure 1.** Smooth curve fittings between RDW-CV(%) and admission RBC count (*10^12^/L).**





Note:

The data for analysis were derived from one of the five multiply-imputed datasets.

1. Analysis based on all included neonates (N=803). The *P* for overall association was 0.002, and the *P* for non-linearity was 0.173.

(B) Analysis using data within the 0.5th to 99.5th percentiles of RDW-CV to reduce the impact of extreme values. The *P* for overall association was 0.190, and the *P* for non-linearity was 0.808.

In both panels, the red solid line indicates the estimated linear regression, and the yellow shaded band represents the 95% confidence interval. The blue histogram shows the distribution of RDW-CV values. The linear regression model was adjusted for neonatal sex, birth weight, admission weight, age at admission, age at admission blood draw, maternal age, gestational week, pre-pregnancy BMI, delivery mode, WBC, HGB, PLT and HDN.

**Supplementary Table 1. Multivariable linear regression analyses of the associations between RDW-CV and **bilirubin decline velocity during treatment in NHB.****

|  | N .total | Non-Adjusted Model *β* (95%CI) | *P* | Model Ⅰ *β* (95%CI) | *P* | Model Ⅱ *β* (95%CI) | *P* | Model Ⅲ *β* (95%CI) | *P* | Model Ⅳ *β* (95%CI) | *P* |
| --- | --- | --- | --- | --- | --- | --- | --- | --- | --- | --- | --- |
| RDW-CV, % | 803 | -2.68 (-4.02~-1.35) | <0.001 | -2.38 (-3.68~-1.09) | <0.001 | -2.50 (-3.82~-1.19) | <0.001 | -2.57 (-3.90~-1.24) | <0.001 | -2.27 (-3.60~-0.94) | 0.001 |
| RDW-CV (11.9% - <14.6%) | 261 | 0 (Ref) |  | 0 (Ref) |  | 0 (Ref) |  | 0 (Ref) |  | 0 (Ref) |  |
| RDW-CV (14.6% - <15.2%) | 260 | -2.28 (-5.50~0.94) | 0.165 | -1.95 (-5.01~1.12) | 0.214 | -1.66 (-4.73~1.41) | 0.290 | -1.72 (-4.80~1.36) | 0.275 | -1.60 (-4.66~1.46) | 0.306 |
| RDW-CV (15.2%-20.8%) | 282 | -5.64 (-8.80~-2.48) | 0.001 | -5.24 (-8.28~-2.20) | 0.001 | -5.21 (-8.29~-2.14) | 0.001 | -5.33 (-8.44~-2.21) | 0.001 | -4.68 (-7.79~-1.57) | 0.003 |
| *P* for trend | 803 |  | 0.001 |  | 0.001 |  | 0.001 |  | 0.001 |  | 0.003 |

Note: Multivariable **linear regression analysis of RDW-CV as a continuous variable with bilirubin decline velocity (μmol/(L·d)) in NHB.**

The data for analysis were derived from one of the five multiply-imputed datasets.

Non-Adjusted Model: No covariates were adjusted.

Model I: Adjusted for neonatal sex, birth weight, admission weight, neonatal blood type, a**ge at admission, age at admission blood draw**.

Model II: Adjusted for model I + maternal age, **gestational week**, **pre-pregnancy** BMI, and delivery mode.

Model III: Adjusted for model II + WBC, HGB, PLT.

Model Ⅳ: Adjusted for model III +HDN.

**Supplementary Table 2. Multivariable linear regression analyses of the associations between RDW-CV and **RBC** count**.****

|  | N.total | Non-Adjusted Model *β* (95%CI) | *P* | Model Ⅰ *β* (95%CI) | *P* | Model Ⅱ *β* (95%CI) | *P* | Model Ⅲ *β* (95%CI) | *P* | Model Ⅳ *β* (95%CI) | *P* |
| --- | --- | --- | --- | --- | --- | --- | --- | --- | --- | --- | --- |
| RDW-CV | 803 | 0.08 (0.04~0.12) | <0.001 | 0.07 (0.03~0.11) | 0.001 | 0.08 (0.04~0.12) | <0.001 | 0.03 (0.01~0.05) | 0.001 | 0.03 (0.01~0.05) | 0.001 |

Note: Multivariable **linear regression analysis of RDW-CV as a continuous variable with RBC.**

The data for analysis were derived from one of the five multiply-imputed datasets.

Non-Adjusted Model: No covariates were adjusted.

Model I: Adjusted for neonatal sex, birth weight, admission weight, neonatal blood type, a**ge at admission, age at admission blood draw**.

Model II: Adjusted for model I + maternal age, **gestational week**, **pre-pregnancy** BMI, and delivery mode.

Model III: Adjusted for model II + WBC, HGB, PLT.

Model Ⅳ: Adjusted for model III +HDN.
