# Supplementary figures and images for: Association between red blood cell distribution width coefficient of variation and post-treatment bilirubin decline velocity in neonatal hyperbilirubinemia
Source: Front Pediatr. 2025 Dec 4;13:1690164. doi: 10.3389/fped.2025.1690164 (PMC12711753; doi:10.3389/fped.2025.1690164)

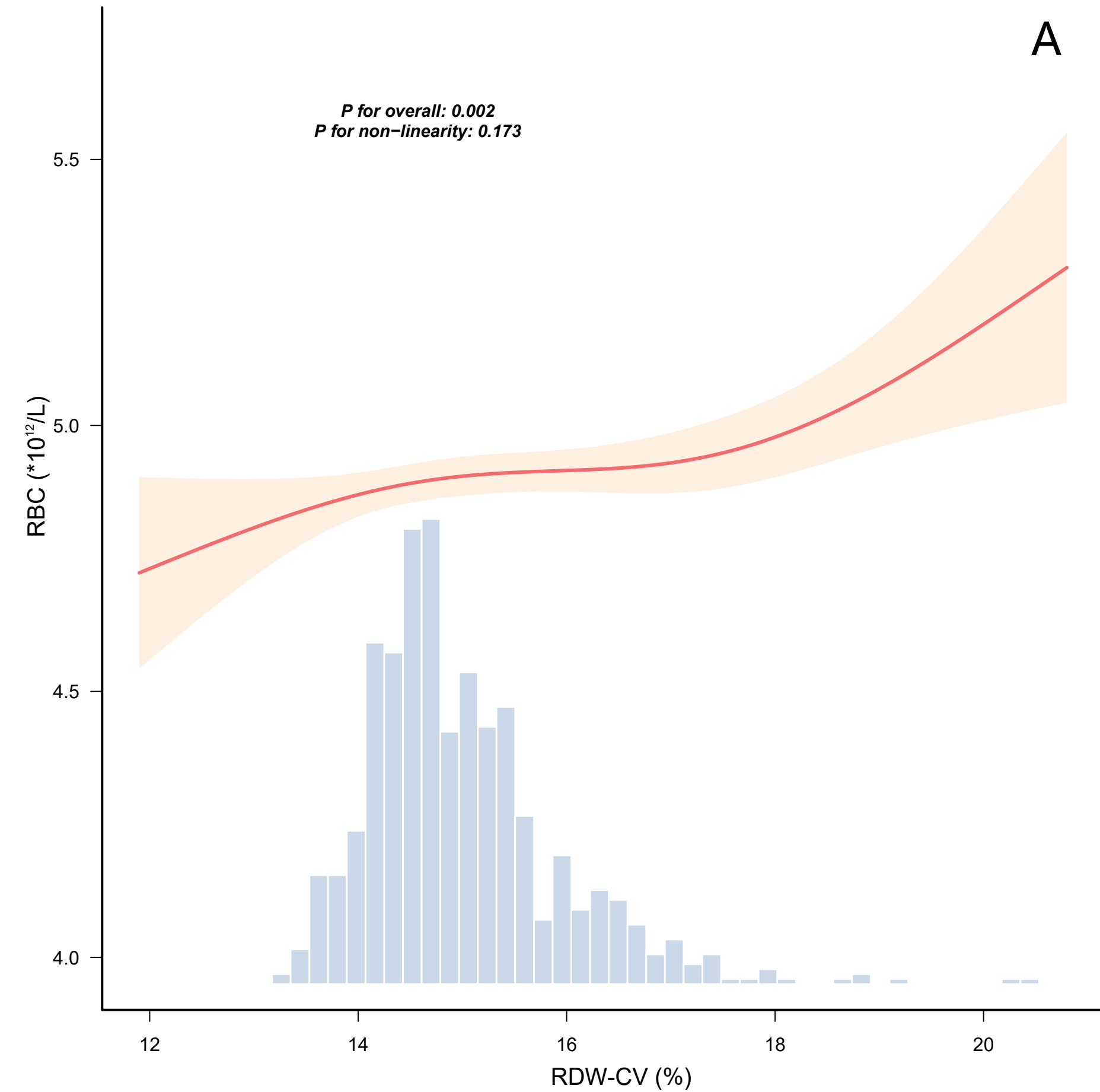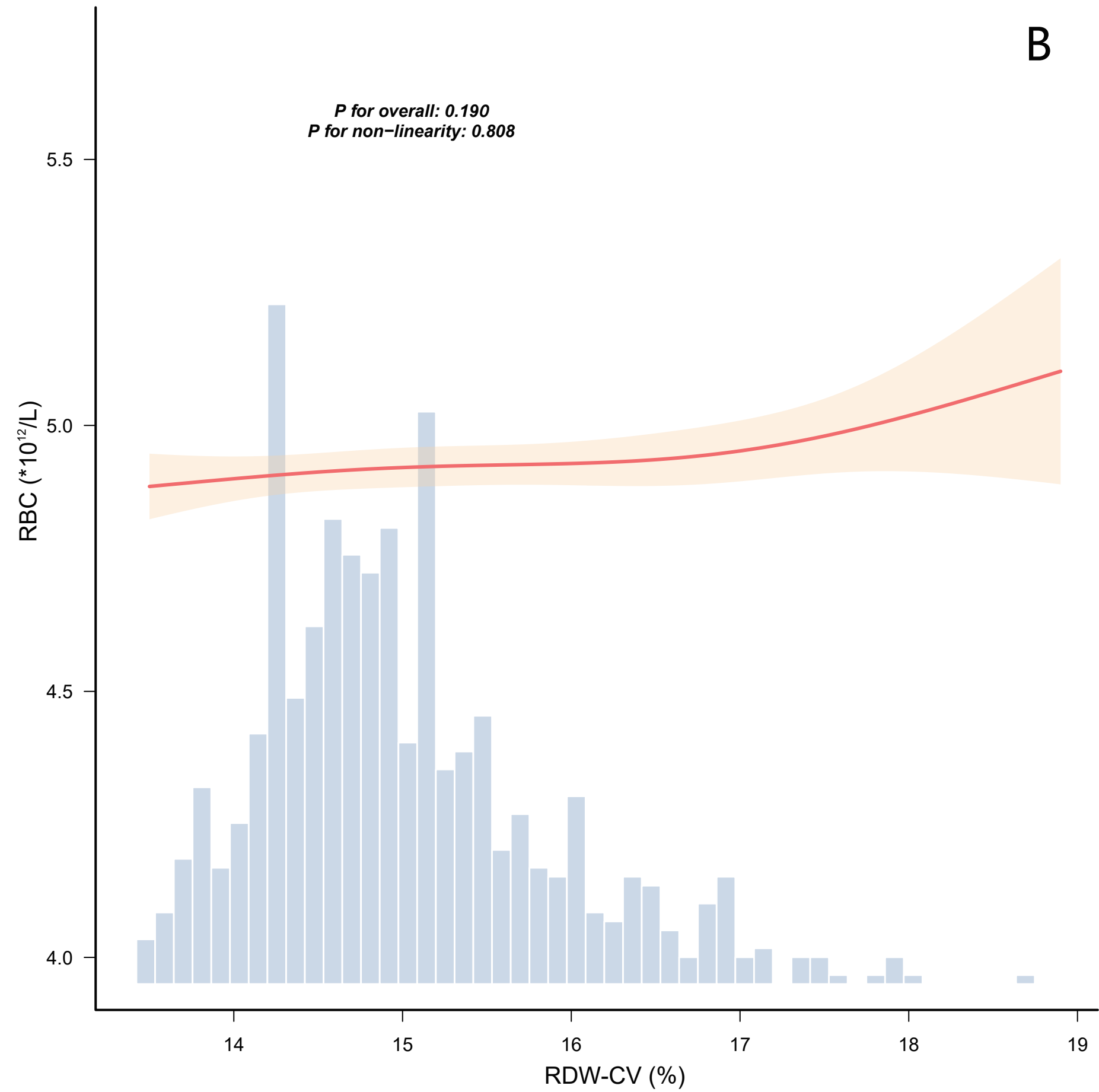

Supplement: Supplementary file 2 [file DataSheet1.pdf]
